# ‘Walk This Way’ – a pilot of a health coaching intervention to reduce sedentary behaviour and increase low intensity exercise in people with serious mental illness: study protocol for a randomised controlled trial

**DOI:** 10.1186/s13063-016-1660-2

**Published:** 2016-12-12

**Authors:** Julie Williams, Brendon Stubbs, Fiona Gaughran, Tom Craig

**Affiliations:** 1 Health Service and Population Research Department, Institute of Psychiatry, Psychology and Neuroscience, King’s College London, London, SE5 8AF UK; 2 The Collaboration for Leadership in Applied Health Research and Care (CLAHRC), South London, UK; 3 Physiotherapy Department, South London and Maudsley NHS Foundation Trust, Denmark Hill, London, SE5 8AZ UK; 4 Psychosis Studies, Institute of Psychiatry, Psychology and Neuroscience, King’s College London, London, UK; 5 National Psychosis Service, South London and Maudsley NHS Foundation Trust, London, UK

**Keywords:** Sedentary behaviour, Physical activity, Serious mental illness, Psychosis, Metabolic syndrome

## Abstract

**Background:**

People with serious mental illness (SMI) (psychosis, bipolar disorder and major depressive disorder) experience a considerable risk of premature mortality because of cardiovascular disease. Recent research has demonstrated that this population spends almost 13 h per day being sedentary. Sedentary behaviour is an independent risk factor for cardiovascular disease and mortality. Given the potential for physical activity to improve health and well-being in people with SMI, we developed a pilot randomised controlled trial (RCT) to evaluate a coaching intervention aimed at reducing sedentary behaviour and increasing physical activity in people with SMI. Our primary aim was to assess the acceptability and feasibility of the intervention. Secondary aims were to see if the Walk This Way (WTW) intervention decreased sedentary behaviour and increased activity levels.

**Methods/design:**

People with SMI who met any of the following criteria were recruited by two community mental health teams in South London: (1) overweight, (2) at risk for or have diabetes, (3) smoke tobacco or (4) have a sedentary lifestyle. Care co-coordinators (clinical case managers) identified potentially eligible participants within their caseload, and these individuals were subsequently invited to participate. All participants’ physical activity (self-reported and accelerometer-recorded), health status (including metabolic blood tests) and motivation to exercise were assessed at baseline. Participants were randomised to receive treatment as usual or the WTW intervention. WTW consisted of an educational intervention at baseline on the benefits of an active lifestyle. Participants were then given a pedometer and received fortnightly coaching from a staff member trained in coaching skills to help them to set daily walking targets, and they were invited to a weekly walking group. The WTW intervention lasted 17 weeks in total.

**Discussion:**

To our knowledge, WTW is the first RCT to investigate the impact of a health coaching intervention targeting sedentary behaviour in people with SMI. It is hoped that if the intervention is feasible and acceptable, further large scale study can be developed and implemented in routine care.

**Trial registration:**

ISRCTN37724980. Registered on 4 Aug 2015.

## Background

People with serious mental illness (SMI) experience a mortality rate two to three times higher than that of the general population [1, 2]. This mortality gap translates to a 10- to 20-year shortened life expectancy and appears to be widening [2, 3]. Approximately 60 % of the excess mortality observed in SMI is due to physical co-morbidities, predominantly cardiovascular diseases (CVDs) [3]. The authors of a recent large-scale meta-analysis of 198 studies found that 32.6 % of people with SMI have metabolic syndrome (MetS), which is highly predictive of CVD [4]. Cardiometabolic abnormalities appear to be particularly prevalent among people in England with SMI. This is exemplified by a recent U.K. study of 450 participants with SMI who were using mental health services in which researchers found that nearly all women and most men had a waist circumference exceeding the International Diabetes Federation (IDF) threshold for central obesity, with the half of the sample being obese, one-fifth meeting the criteria for type 2 diabetes mellitus (T2DM) and 57 % meeting the IDF criteria for MetS [5]. Moreover, people with schizophrenia [6], bipolar disorder [7] and major depressive disorder [8] are at significantly increased risk of T2DM. Much of the increase in CVD and MetS appears to be related to anti-psychotic medication and unhealthy lifestyle factors [9].

There is a plethora of literature demonstrating that sedentary behaviour is associated with an increased risk of T2DM, CVD and mortality in the general population [10]. Recently, the World Health Organisation (WHO) stated that physical inactivity is the fourth leading global cause of avoidable mortality [11], and Public Health England (PHE) have made calls for everybody to be active every day [12]. Given the deleterious impact of a sedentary lifestyle, both the WHO and PHE now stipulate that preventing physical inactivity is of equal importance with encouraging smoking cessation. The authors of a recent meta-analysis demonstrated that people with psychosis spend almost 13 h per day being sedentary, equating to 3 h per day more than control subjects [13]. The levels of sedentary behaviour reported in this meta-analysis are among the highest recorded in any population. Research regarding the harmful effects of sedentary behaviour among people with SMI is sparse, but there is good reason to believe that this may be particularly marked in people with SMI. A recent study [14] demonstrated that higher levels of sedentary behaviour is predictive of heightened C-reactive protein (CRP) levels, a key predictor for increased CVD risk.

Regarding the general population, meta-analyses of randomised controlled trials (RCTs) have demonstrated that physical activity is potentially as effective as pharmacological interventions in reducing cardiovascular mortality [15]. Moreover, a recent meta-analysis established that physical activity can improve anthropometric measurements, psychiatric and depressive symptoms in people with SMI [16]. However, people with SMI experience a range of barriers to physical activity and struggle to meet recommended target guidelines such as 150 minutes of moderate physical activity per week. In general medicine, there have recently been calls to place an emphasis on reducing sedentary behaviour and replace this with light physical activity for groups that frequently do not meet recommended physical activity targets, because some physical activity is better than none [17]. Recently, a similar call was made to consider this approach in people with SMI [18].

In order to overcome multiple barriers, there is a need to provide innovative, simple and enjoyable ways for people with SMI to engage in physical activity. In the general population, pedometer-based interventions can increase habitual physical activity participation, decrease body mass index (BMI), decrease systolic blood pressure and possibly improve cholesterol [19, 20]. The authors of a recent systematic review [21] identified ten walking studies (mostly non-RCTs) of people with schizophrenia and concluded that there is promising evidence that walking interventions can reduce weight and positively influence psychiatric symptoms in people with schizophrenia. However, these studies were all short in duration, and no high-quality adequately powered RCT exists, nor does any study exist in the United Kingdom. Walking is a cheap and simple form of physical activity that may be a feasible method to reduce sedentary behaviour and increase habitual physical activity and improve wider health outcomes for people with SMI [21].

Given the potential opportunity to employ walking as a strategy to reduce sedentary behaviour and improve health among people with SMI, we developed a pilot RCT including a pedometer-based coaching intervention to reduce sedentary behaviour and increase habitual physical activity, called *Walk This Way* (WTW). Specifically, WTW was designed to target service users with SMI who may fall into any of the following groups: (1) overweight, (2) at risk for or have T2DM, (3) smoke tobacco or (4) sedentary.

### Aims

The primary aim of this pilot RCT is to establish the acceptability of the WTW intervention to service users by evaluating if participants can be recruited into the study and if they complete the intervention. Secondary aims are to estimate if, compared with treatment as usual (TAU), the WTW intervention (1) reduces sedentary behaviour (reduce the total minutes per day of sedentary behaviour) and (2) increases daily physical activity (light, moderate and vigorous in minutes per day) in people with SMI. In addition, we will consider the impact of the WTW versus TAU on glucose regulation, progression to T2DM and MetS risk factors, including BMI and waist circumference.

## Methods/design

### Design

**Fig. 1:**
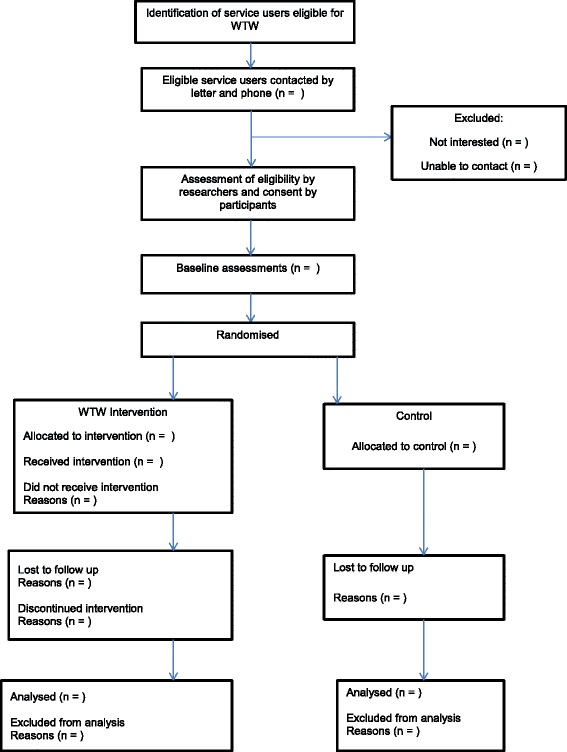
Walk this Way (WTW) study flow diagram

**Fig. 2:**
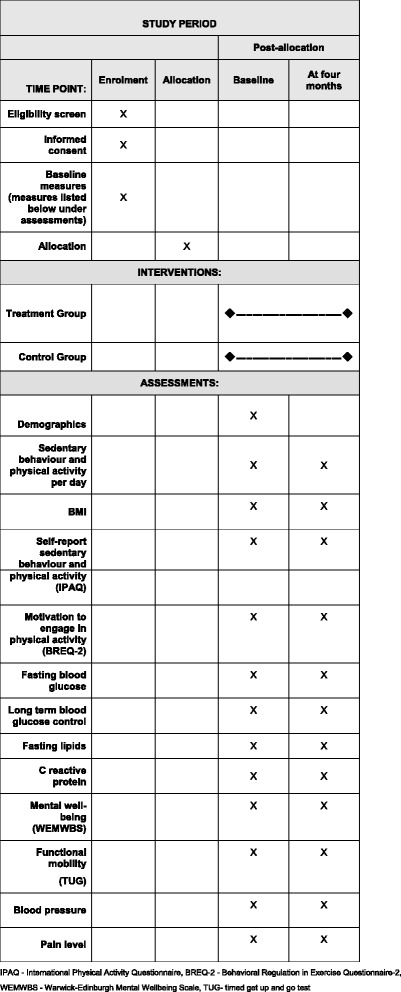
Schedule of enrolment, interventions and assessments. *BMI* Body mass index, International Physical Activity Questionnaire, *BREQ-2* Behavioural Regulation in Exercise Questionnaire-2, *WEMWBS* Warwick-Edinburgh Mental Wellbeing Scale, *TUG* Timed Get Up and Go Test

WTW is a 17-week pilot RCT programme that includes an initial group education session, fortnightly coaching sessions, and the provision of a pedometer and calendar to enable participants to monitor their daily physical activity. Participants assigned to the TAU arm will receive brief information on the benefits of being more active and will be given information on local walking routes of interest. Ethical approval for the trial has been obtained from the City Road and Hammersmith National Research Ethics Service Committee (15/LO/1188), and the trial has been registered in the ISRTCN Registry (ISRCTN37724980). A flow diagram of the WTW study is shown in Fig. 1, and the schedule of enrolment, interventions and assessments is provided in Fig. 2.

### Setting

The WTW intervention will take place in one location within the South London and Maudsley NHS Foundation Trust (SLaM). The team at the location has experience with running healthy lifestyle programmes for people with SMI.

### Participants

Participants will be recruited from among community teams in SLaM. The following eligibility criteria will be used:

1. Diagnosis of any SMI (schizophrenia, psychosis, bipolar disorder and major depression)
2. Meeting any one of the following criteria as determined by a care co-ordinator:
  - Overweight
  - At risk for or have T2DM
  - In the clinician’s view, have a sedentary lifestyle
  - Smoke tobacco
3. Ability to provide informed consent
4. Ability to understand English

We aim to recruit 40 participants, 20 in the intervention group and 20 in the control group. This number is based on recruiting a big-enough sample to assess the feasibility of the study and on the resources available [22].

### Procedure

Researchers will work with the care co-ordinators within each referring team to identify service users who meet the eligibility criteria. Care co-ordinators will be asked to identify any reasons why we should not contact people (such as they are very unwell either physically or mentally). All service users who meet our criteria, and where there are no reasons not to contact them, will be sent a letter explaining the study. This letter will be followed by a telephone call 1 week later.

We will record the details of the service users who (1) meet our criteria, (2) who contact us once they have received the letter, (3) agree to meet a researcher when contacted by us and (4) decide they do not want to meet a researcher when contacted. This level of detail will help us to assess the acceptability of the intervention when described to service users. We will then record details of who takes part in the research and the rates of drop-out from the intervention.

All service users who express an interest will be invited to meet a researcher, who will explain the study and ask if they would like to participate. They will be given written information on the study, along with the opportunity to ask any questions. We will give people 24 h to decide if they would like to participate. If they are happy with the information they receive, they will be asked to give informed consent and baseline measures will be completed. The participant will then be asked to wear an accelerometer for 4 days. Each participant will be reimbursed £10 for the baseline paper measures and £10 for wearing the accelerometer. After completion of the baseline measures, the participants will be informed of their allocation status.

### Data collection

#### Primary outcome: acceptability, feasibility and recruitment rates of the study

We will measure (1) whether it was possible to recruit sufficient participants into the study within a particular time frame and (2) how many people who were recruited into the study completed the intervention.

#### Secondary outcomes

Sedentary behaviour and physical activity time per day will be recorded. All participants will be required to wear a wrist-worn GENEActiv accelerometer (GENEActiv, Kimbolton, UK) for at least 4 days (including 1 weekend day if possible) at baseline that will measure habitual sedentary behaviour and walking activity each day. The accelerometer will record how many minutes per day each participant is sedentary and engages in light, moderate and vigorous physical activities. A recording is made of each 60-second period (called an ‘epoch’), and this is classified as being either sedentary or light, moderate or vigorous physical activity. The cut-off points were defined according to metabolic equivalents (METs) of sedentary (<1.5 METs), light (1.5–3.99 METs), moderate (4.00–6.99 METs) and vigorous (>7+ METs), based on standardised algorithms with high sensitivity and specificity with oxygen consumption [23]. For this study, we are interested in the total minutes of sedentary behaviour per day, number of disruptions in sedentary behaviour and total time spent in physical activity (minutes per day in light, moderate and vigorous activity). Specifically, we are interested in the change in average minutes of sedentary behaviour and physical activity before and after the WTW intervention. We will measure if the WTW intervention group changes pre- and post-intervention in sedentary behaviour and physical activity and also measure if this differs from the control group. The following outcomes will also be measured at both baseline and follow-up:

1. *Anthropometric measures*: We will measure BMI according to the IDF criteria [24], including height, weight and waist circumference.
2. *Self-report sedentary behaviour and physical activity*: The International Physical Activity Questionnaire (IPAQ) [25] will be used to capture self-reported physical activity and sedentary behaviour. The IPAQ has questions asking about the amount of vigorous and moderate activity done in the last 7 days, how often the person has walked for more than 10 minutes in the last 7 days, and how many hours the person is sedentary on a usual day.
3. *Motivation to engage in physical activity*: The Behavioural Regulation in Exercise Questionnaire-2 (BREQ-2) [26] is a 19-item interviewer-administered questionnaire designed to consider an individual’s motivation towards exercise. The BREQ-2 has been validated in people with schizophrenia [27].
4. *Blood samples*: We will collect information from blood samples taken by a phlebotomist. This information will include fasting blood glucose, insulin (to calculate the homeostasis model assessment of estimated insulin resistance index), long-term blood glucose control (as measured by glycated haemoglobin), fasting lipids and CRP.
5. *Mental well-being*: We will use the Warwick-Edinburgh Mental Wellbeing Scale [28], a 14-item self-report measure, to assess well-being. Respondents rate their experience regarding each statement over the last 2 weeks. Each item is scored using a 5-point Likert scale ranging from 1 (none of the time) to 5 (all of the time), with the total score ranging from 14 (low well-being) to 70.
6. *Functional mobility*: We will ask all participants to complete the Timed Get Up and Go (TUG) Test [29]. The TUG Test requires participants to stand up from a chair, walk 3 meters, turn around, walk back, and sit down again. The time taken is measured in seconds, and scores represent functional mobility, with higher scores indicating increasing mobility difficulties. Times longer than 13.5 seconds are predictive of falls in the general older adult population.

We will also collect details of age, gender, ethnicity, living arrangements, pain level, smoking status, psychiatric diagnosis and current medications.

### Randomisation

The randomisation will be done by a researcher independent of the study using the random sequence generator in random.org. The researchers will not know which arm the participant has been consigned to before the baseline assessment. Participants will be told if they have been randomised to either the intervention and control group after the baseline assessment.

### Walk This Way intervention

#### Initial group education session

Participants assigned to the WTW intervention will attend a baseline educational group session which will include a minimum of five and a maximum of ten people, depending on the rate of recruitment, and is amended from the Walk, Address sensations, Learn about exercise, Cue exercise behaviour for persons with schizophrenia spectrum disorders (‘WALC-S’) programme [30, 31], which is a motivational intervention based upon the self-efficacy theory. The aim of the sessions will be to introduce the basics of the benefits of walking for exercise and why exercise is beneficial, as well as to give information, support and motivation to help participants to independently walk more in their daily routines. In the group sessions, we will also introduce the concept of sedentary behaviour and the harms and strategies to sit less and move more, including disrupting prolonged periods of sitting. At the educational session, researchers will have information on the participants’ habitual levels of physical activity obtained from baseline data collection. The group session will also include goal setting, in which participants will be encouraged to set their own daily walking targets to increase their habitual levels of walking. All participants will be given a pedometer to self-monitor how far they walk throughout the intervention on a daily basis. Specifically, participants will be given a Yamax Digi-Walker CW-700 pedometer (Yamasa Tokei Keiki Co., Ltd., Tokyo, Japan), which is the criterion pedometer with optimal accuracy. It provides direct step-count feedback to participants, and participants will be encouraged to self-monitor their daily activity levels and record them on an individualised calendar.

The principles of coaching will also be presented during the education session, and the participants will be introduced to their ‘coach’. Finally, all participants will go on a walk to ensure they are able to operate their pedometers.

#### Continuing support and coaching

Participants will meet with an assigned worker (i.e., their coach) every 2 weeks. The participant and coach will review the participant’s walking calendar and address any barriers to and facilitators of engaging in physical activity and reducing sedentary behaviour. The participant’s daily walking target (number of steps per day) will be reviewed and amended as required by setting SMART (Specific, Measurable, Attainable, Realistic and Timely) goals with the patient. The brief meetings (lasting 20–30 minutes) every 2 weeks will provide an opportunity for the worker to provide motivational support to assist the participant to reach his or her daily step goals. If the worker identifies barriers such as inappropriate footwear, then the worker will be given funds to purchase appropriate footwear for the participant. The staff doing the coaching will have training in goal attainment and coaching skills because this has been found to increase motivation and adherence [32]. Coaching is being used more often in mental health services, and a recent study of coaching to reduce obesity in people with SMI had encouraging results [33]. Coaching sessions will be audiotaped to assess fidelity, and the coaches will have regular supervision sessions with a coaching specialist.

In addition, the worker will arrange for all members who attended the group educational session to continue to meet every week for a regular group walk. This will provide an element of social support but will remain optional for the participants.

### Control condition

Participants in the control group will complete baseline measures, and then they will receive written information on the benefits of increasing activity levels. This advice will be given in accordance with NHS Foundation Trust policy on physical health.

### Follow-up assessment

The follow-up assessment will be undertaken at the end of the intervention. At follow-up, all measures will be repeated (apart from sociodemographic information). Each participant will be paid £10 for the paper measures and £10 for wearing the accelerometer. These assessments will be done by a research worker who is blind to allocation status. We will attempt to follow all participants, including those who discontinue the intervention early.

### Process evaluation

In addition to the outcome assessment, we will also undertake a qualitative process evaluation to find out (1) how participants experienced the intervention, if there are any parts of the intervention that need to be changed, and what factors influenced people in completing/not completing the intervention; and (2) how the staff doing the coaching intervention experienced this. We will also use this process evaluation to ask service users about the issues they have regarding their physical health and what support they would like with this.

### Data management

Each participant will be given an identification number. All information collected will be kept confidential; all identifiable data will be kept in a locked cabinet; and forms with identifiable data will be kept separate from the outcome data. The outcome data will be collected on paper forms, and the data will then be transferred to an IBM SPSS database (IBM, Armonk, NY, USA). Data quality will be enforced by having range checks, valid values and data double-entry. We will collect all information on instances of adverse events, including hospitalisation for either mental or physical problems, as well as any mental health relapse or home treatment team involvement or accident and emergency visit. Any changes to the protocol will be reported to the research ethics committee. The final dataset will be accessed by the principal investigator and the research team. The protocol, the anonymised participant level dataset and any statistical codes used will be made available on request.

### Analysis

For the primary aim and to evaluate feasibility, we will calculate the percentage of people approached who participate in the intervention and the percentage who complete the intervention. We will use the process evaluation to understand how the intervention was experienced by participants and any changes that they think would improve the intervention.

To evaluate the effectiveness of the intervention, we will investigate changes in pre- and post-test scores for the secondary outcome measure (total minutes of sedentary behaviour per day and total minutes of physical activity) of the WTW participants. We will also investigate changes in the pre- and post-test scores for the other outcome measures. We will compare the mean changes in sedentary behaviour and physical activity (light, moderate and vigorous) in the WTW group versus the control group.

## Discussion

This pilot aims to evaluate the feasibility and acceptability of an intervention to support people with SMI using secondary mental health services to increase their level of activity. If WTW proves to be acceptable, we plan to undertake a larger RCT to assess the effectiveness of such an intervention.

The physical health of people with SMI has become a priority for both primary and secondary services in recent years to address the identified mortality gap. Improving the physical health of people with SMI will require a multifaceted approach that includes the identification of physical health risks to ensure effective treatment is given and interventions are initiated to reduce physical health risks. This is happening at a time when there is a growing awareness of the risks of sedentary behaviour in the general population which is leading to approaches to reduce sedentary behaviour in all.

National Institute for Health and Care Excellence guidelines for people with schizophrenia recommend that interventions be offered to support people with SMI to be more active [34], but there are few evidence-based interventions which clinicians can offer. An intervention that is found to be acceptable to people with SMI would be a welcome addition to the field.

## Trial status

The trial is ongoing and no longer recruiting.

## Abbreviations

BMI: Body mass index
BREQ-2: Behavioural Regulation in Exercise Questionnaire-2
CRP: C-reactive protein
CVD: Cardiovascular disease
IDF: International Diabetes Federation
IPAQ: International Physical Activity Questionnaire
MDD: Major depressive disorder
MET: Metabolic equivalent
MetS: Metabolic syndrome
PHE: Public Health England
RCT: Randomised controlled trial
SLaM: South London and Maudsley NHS Foundation Trust
SMI: Serious mental illness
T2DM: Type 2 diabetes mellitus
TAU: Treatment as usual
TUG: Timed Up and Go Test
WALC-S: Walk, Address sensations, Learn about exercise, Cue exercise behaviour for persons with schizophrenia spectrum disorders programme
WEMWBS: Warwick-Edinburgh Mental Wellbeing Scale
WHO: World Health Organisation
WTW: Walk This Way study
